# Stabilization of mismatch repair gene PMS2 by glycogen synthase kinase 3β is implicated in the treatment of cervical carcinoma

**DOI:** 10.1186/1471-2407-10-58

**Published:** 2010-02-23

**Authors:** Yuan Zhang, Yi Min Shu, Shu Fang Wang, Bang Hong Da, Ze Hua Wang, Hua Bin Li

**Affiliations:** 1 Department of Obstetrics and Gynecology, Union Hospital, Tongji Medical College, Huazhong University of Science and Technology, Wuhan 430022, PR China; 2 Allergy and Cancer Center, The First Affiliated Hospital of Sun Yat-sen University, Guangzhou 510080, PR China; 3 Department of Pathology, Baylor College of Medicine, Houston, TX 77030, USA; 4 Department of Medicine, Feinberg Medical School, Northwestern University, Chicago, IL 60611, USA

## Abstract

**Background:**

PMS2 expression loss was reported in a variety of human. However, its importance has not been fully understood in cervical carcinoma. The aim of this study was to determine the expression of PMS2 in cervical carcinoma and evaluate the significance of mismatch repair gene PMS2 regulated by glycogen synthase kinase 3β (GSK-3β) in chemosensitivity.

**Methods:**

We examined PMS2 and phosphorylated GSK-3β(*s*9) expression in cervical carcinoma tissues using immunohistochemical staining. Furthermore, we detected PMS2 expression in HeLa cells and evaluate the interaction with GSK-3β after transfection with GSK-3β by small interference RNA (siRNA), co-immunoprecipitation and immunoblotting. We also evaluated the effect of PMS2 transfection on HeLa cells' chemosensitivity to cisplatin treatment.

**Results:**

We found significant downregulation of PMS2 in cervical carcinoma, which was negatively associated with phosphorylated GSK-3β (*s*9). Furthermore, we demonstrated GSK-3β transfection was able to interact with PMS2 and enhance PMS2 production in HeLa cells, and increased PMS2 production was responsible for enhanced chemosensitivity.

**Conclusions:**

Our results provide the evidence that stabilization of PMS2 production by GSK-3β was important to improve chemosensitization, indicating the significance of GSK-3β-related PMS2 downregulation in the development of cervical carcinoma and in developing a potential strategy for chemotherapy.

## Background

Globally, cervical cancer is second only to breast cancer as the leading cause of cancer death in women, with a prevalence of 2.3 million cases and an annual incidence of half million [1]. And approximately 275,000 women die from this disease every year worldwide [2]. Despite advances in the study of cervical cancer over the past 20 years, its pathogenesis is still not fully understood [3].

Mismatch repair (MMR) is one of the most important DNA repair processes for maintaining genetic fidelity. MMR deficiency leads to carcinogenesis through increased mutation frequency, loss of cell cycle arrest, and decreased apoptosis in response to DNA damage. In addition to MSH2 and MLH1, other human mismatch repair genes including MSH3, MSH6, MLH3, PMS1, and PMS2 have been identified [4]. To date, a disrupted MMR system has been identified in several cancers, including hereditary nonpolyposis colorectal cancer (HNPCC), and some sporadic cancers [5]. The bulk of germline HNPCC mutations, roughly 90%, resides in two MMR genes, MSH2 and MLH1, with mutations in MSH6 (7-10%) associated with atypical HNPCC [6]. However, PMS2 mutations are quite rare in HNPCC as well as various other cancers [7-9].

It is very important to recognize that aberrant expression of MMR proteins has been reported to be associated with increased risk of cervical cancer [10], low and loss of expression of MMR in patients with cervical cancer has been described by different groups as well. For instance, Chung et al. showed loss of MSH2 expression in 7 of 50 squamous cervical cancers [11], and Giarnieri et al. showed loss of MSH2 in 10 of 23 cervical cancers [12]. Ciavattini et al. recently found MSH2 and MLH1 expression to be lower in 28 invasive squamous cervical cancers compared to cervical intraepithelial lesions [13]. These findings therefore implied that MMR may be also associated with the pathogenesis of cervical cancer. The PMS2 gene encodes a MutL-homologous protein that forms a heterodimer with MLH1, and the resulting complex, MutLα, interacts with MutSα/β to activate MMR. Because monomeric PMS2 is highly unstable [14], and the reported mutation rate of PMS2 is quite low in various cancers, we hypothesized that certain types of exogenous DNA-damaging agents may rapidly degrade PMS2 production and disrupt the MMR system for facilitating carcinogenesis. Given that few data are available regarding PMS2 modulation in human cervical carcinoma, investigating the mechanisms underlying the regulation of PMS2 may be essential for understanding the significance of PMS2 in carcinogenesis.

To address this issue, we focused on the possibility of modulation of MMR proteins by taking advantage of Wnt signaling activation in this study, since certain key components such as Glycogen synthase kinase 3β (GSK-3β) in Wnt signaling have been implicated in a number of cancers [15]. GSK-3β is active in resting cells, and it is modulated by site-specific phosphorylation at the site of *s*9 (inactivation) during cellular responses. We chose GSK-3β and PSM2 in this study mainly because germline PMS2 mutations are rarely reported, unlike other components. On the other hand, our preliminary findings demonstrated a consensus motif of VSSSH GPSDP TDRAE in PMS2 (*s*499) by gene sequencing. This consensus motif was shown to be the common phosphorylation motif recognized and activated by GSK-3β in previous studies [16]. Our preliminary finding thus provides the possibility that the interaction of GSK-3β with the PMS2 consensus motif contributes to maintaining genomic stability and promoting protective responses of mammalian cells to exogenous DNA damage (e.g., apoptosis). If so, the rapid induction and degradation of PMS2 may be essential in the balance between cell survival and death as a sensor of environmental changes in cervical carcinoma.

To verify this hypothesis, we examined the expression of PSM2 protein in cervical cancer in this study, evaluated the effects of cisplatin on PMS2 expression in HeLa cells, and examined the importance of GSK-3β binding on PMS2 stability and chemosensitization to cisplatin in HeLa cells by taking advantage of co-immunoprecipitation (IP), small interference RNA (siRNA) and flow cytometric analysis. Our finding therefore will be helpful in clarifying the molecular mechanisms involving the modulation of MMR proteins by Wnt signaling in the development of cervical carcinoma and in developing a possible therapeutic target for future chemotherapy.

## Methods

### Materials

3-(4,5-Dimethylthiazol-2-yl)-2,5-diphenyltetrazolium bromide (MTT), cisplatin (cis-diammine-dichloroplatinum), dimethyl sulfoxide (DMSO), and lithium chloride (LiCl), a GSK-3β inhibitor, were purchased from Sigma. Human pGSK-3β (*s*9) and β-actin antibody were obtained from Santa Cruz Biotech. MLH1, MSH2, and PSM2 antibody was obtained from BD Biosciences. The plasmids pCGN-GSK-3β (WT), pGEX-GSK-3β, constitutively active GSK-3β (GSK-3β-CA; S9A GSK-3β), kinase-dead GSK-3β (GSK-3β-KD; pCGN-GSK-3β-KD) plasmids and pCGN-PMS2 plasmids were kindly provided by Dr. Qingqing Ding (MD Anderson Cancer Center, Houston, USA). Plasmid vector GSK-3β siRNA (pKD-GSK-3β-V1;Si-GSk-3β) was purchased from Upstate Biotechnology. Enhanced chemiluminescence western blotting detection reagents were purchased from Cell Signaling. Transfection reagents (Lipofectamine 2000 and PLUS reagent) were purchased from Invitrogen Corporation.

### Immunohistochemical staining

For immunohistochemical staining, seventy-eight surgically resected human cervical carcinoma tissues were collected from the Departments of Obstetrics and Gynecology, Wuhan Union Hospital, and the study was approved by the institutional review board. Immunohistochemistry was performed according to our previously described protocol [17]. Briefly, human tumor tissues embedded in paraffin were cut into 5-μm sections and placed onto glass slides. After antigen retrieval, sections were stained for the expression of MLH1 (1:50), MSH2 (1:50), PMS2 (1:100), and pGSK-3β (1:100), and then detected by streptavidin-biotin-horseradish peroxidase complex formation. Tumor sections stained by isotype matched IgG instead of primary antibodies were used as a negative control. The immunostaining was considered positive when the tumour mass occupied more than 10% of the cross-sectional core area and when 10% or more of the neoplastic cells were stained.

### Cell culture

Human cervical carcinoma HeLa cell lines (American Type Culture Collection) were cultured in RPMI 1640 supplemented with 10% fetal calf serum (FCS) (Invitrogen), penicillin (100 units/mL), and streptomycin (100 μg/mL; Invitrogen). All cultures were maintained at 37°C in a humidified atmosphere with 5% CO_2_.

### Transient transfection, RNA interference, and cisplatin treatment

For plasmid transfection, HeLa cells were seeded in a 24-well plate at low densities overnight in RPMI 1640 supplemented with 10% FCS. Cells were transiently transfected at 70% to 90% confluency, using the plasmid DNA (up to 4 μg) mixed with the Lipofectamine 2000 reagent (Invitrogen) at the DNA (μg)/lipid (μL) ratio of 1:2.5. Silencing of GSK-3β was similarly performed with Si-GSk-3β plasmids in HeLa cells. At 4-6 h post-transfection, the plasmid- or siRNA-containing medium was replaced with normal culture medium containing 10% FCS, and the cells were incubated in a 5% CO_2 _incubator at 37°C. Transfected cells were then cultured in fresh medium for up to 12 to 48 h and harvested for gene expression and other assays. For specific blockage of GSK-3β activity, LiCl (20 mM) was added to HeLa cells simultaneously. For cisplatin treatment, various concentrations (0-10 μg/ml) of cisplatin were added to HeLa cells. DMSO alone and medium alone served as controls. Time point chosen for cisplatin addition to the transfected cells was 24 h after transfection, based on preliminary experiments.

### Assessment of cell viability by MTT Assay

Treated or untreated cells were seeded into 96-well plates at 1 × 10^3 ^cells per well overnight and then incubated with different concentrations of cisplatin (from 0 to 10 μg/ml). After culture for 24 h, 20 μl of MTT dye solution (5 mg/ml) was added to each well, and samples were incubated at 37°C for 4 h. The formazan product was dissolved by adding 200 μL of DMSO to each well, and the plates were read at 570 nm. Cell growth activity was determined according to the formula for relative cell viability (OD_570 _from treated cells/OD_570 _from untreated cells ×100%). All measurements were done in triplicate, and the results were calculated from three independent experiments.

To determine the cytotoxicity of cisplatin and combination treatments of cisplatin and GSK-3β WT plasmid, HeLa cells were seeded onto 96-well plates at a density of 1 × 10^3 ^cells/well. Twenty-four hours later, the cells were transfected with GSK-3β WT plasmid at different concentrations (0, 1, 2, and 4 μg/ml). Cells treated with PBS were used as a control. Cisplatin at various concentrations (0-10 μg/ml) was also added to each well. The cells were incubated for 24 h, and cell growth and viability were analyzed by the MTT assay.

The effects of the combination treatment of PMS2 plasmid and cisplatin were analyzed with CalcuSyn software (Biosoft, Cambridge, UK) to determine the combination index (CI). Generally, it is considered that a CI <1.0 indicates synergism, CI = 1.0 indicates an additive effect, and CI >1.0 indicates antagonism.

### Semi-quantitative RT-PCR analysis for PSM2 mRNA levels

Total RNA was isolated from HeLa cells after treatment with cisplatin (10 μg/ml), using TRIzol reagent (Invitrogen) according to the manufacturer's instructions. Total RNA (2 μg) was reverse transcribed using M-MuLV Reverse Transcriptase (Promega) and oligo(dT)18 as primer. cDNA was amplified by PCR using Taq DNA polymerase (Promega). The following primers were designed for RT-PCR: PSM2 forward 5'-GAG AAC CTG CTA AGG CCA TC-3', PSM2 reverse 5'-ATG GTG ACA TCG CTC AGT GC-3' (product size, 340 bp); β-actin forward 5'-ATC TGG CAC CAC ACC TTC CA-3' and reverse 5'-CTC CTT AAT GTC ACG CAC GA-3'(product size, 476 bp). All the primers were synthesized by Invitrogen. The thermocycling conditions were as follows: 30 s at 94°C, 45 s at 56°C, 30 s at 72°C, and 30 cycles followed by extension for 7 min at 72°C. PCR products were analyzed with 1.5% agarose gel electrophoresis in the presence of ethidium bromide for UV light transilluminator visualization. The relative PSM2 mRNA level was normalized to β-actin with Bio-Rad Quantity One 1-D Analysis Software (Bio-Rad). The experiment was repeated in triplicate.

### Immunoprecipitation and Immunoblotting analysis

Treated and untreated HeLa cells were lysed for 10 min on ice with the same lysis buffer used for immunoblotting. Following centrifugation at 14,000 g for 10 min at 4°C, 1 ml of clear lysates was incubated with 5 μl of rabbit anti-GSK-3β antibody or 5 μl of normal goat IgG overnight with continuous rotation at 4°C. Protein A-sepharose beads (30 μl) were then added, and the samples were gently rocked 4°C for 3 h. After five washes with lysis buffer, the beads were recovered and resuspended in 40 μl of 2× SDS sample buffer (4% SDS, 0.125 mol/l Tris-HCl, 20% glycerol, and 0.04% bromphenol blue, pH 6.8) and then boiled for 5 min. The co-immunoprecipitation (IP) proteins dissociated from the beads were used for immunoblotting analysis.

Immunoblotting was done as previously described [18]. Briefly, Treated and untreated HeLa cells were washed in cold PBS three times, and total cellular protein was extracted in 100 μl of RIPA lysis buffer (Pierce) at 4°C for 30 min. The protein concentration was determined by the Bradford method (Pierce). Samples containing 10 μg of protein were boiled and subjected to sodium dodecyl sulfate polyacrylamide gel electrophoresis (SDS-PAGE) on 10% Tris-glycine gels and transferred electrophoretically to a polyvinylidene fluoride membrane. Primary antibodies (mouse anti-human PSM2, rabbit anti-human GSK-3β, and β-actin monoclonal antibody, 1:2,000) were used, followed by horseradish peroxidase-linked secondary antibody (goat anti-mouse IgG, 1:1,000) and visualized by Enhanced Chemiluminescence kits (Cell signaling). The relative density of PSM2 or GSK3β to β-actin was quantified with Bio-Rad Quantity One 1-D Analysis Software (Bio-Rad). The experiment was repeated in triplicate.

### Immunofluorescence staining

Treated and untreated HeLa cells in an eight-well Lab-Tek II Chamber Slide System (Nalge Nunc Int.; 70% confluence) were fixed with 4% paraformaldehyde in PBS followed by incubation in 0.5% Triton X-100 in PBS. After several washes, cells were blocked with 3% bovine serum albumin in PBS followed by incubation with mouse anti-PSM-2 (1:100) and rabbit anti-GSK-3β (1:100) antibodies at 4°C overnight in a humidified chamber. The cells were subsequently exposed to Texas red or FITC-labeled secondary antibodies (Santa Cruz Biotech) (1:200). The labeled cells were detected under a fluorescence microscope. Cells incubated with fluorescein-conjugated secondary antibodies in the absence of primary antibodies served as negative controls.

### Apoptosis assay by flow cytometric analysis

Treated and untreated HeLa cells were stained with annexin-V and PI using a Vybrant Apoptosis Assay Kit (Invitrogen) according to the manufacturer's instructions. Briefly, HeLa cells were seeded onto 24-well plates for 24 h. The cells were then transfected with PMS2 plasmid (2 μg/ml). Various concentrations of cisplatin (0 or 10 μg/ml) were also added to each well. Cells treated with empty plasmid were used as a control. The cells were incubated for 24 h and were used for analyzing apoptosis and caspase-3 activity. For the apoptotic assay, HeLa cells were harvested and washed once with cold PBS. Cell pellets were re-suspended in 100 μL 1× annexin-binding buffer and 5 μL of FITC-annexin-V (component A), and 1 μL of the 100 μg/mL PI working solution was added to each 100 μL of cell suspension. The cells were incubated on ice for 1 h, washed with cold PBS once again, and re-suspended in 300 μL of 1× annexin-binding buffer, and the stained cells were analyzed for apoptosis by flow cytometry soon after staining.

### Determination of caspase-3 activity

The activity of caspase-3 was measured using the caspase-3 activation kit (R&D systems, Inc., Minneapolis, MN), according to the manufacturer's protocol. Similar to the apoptotic assay, HeLa cells were transfected with PMS2 plasmid (2 μg/ml), and cisplatin of various concentrations (0-10 μg/ml) was also added. The cells were incubated for 0-36 h, and total protein in HeLa cells was extracted with RIPA lysis buffer and quantified by the Bradford method. One hundred micrograms of total protein was added to each well in 96-well microtiter plates (Bio-rad) with DEVD-pNA at 37°C for 1-2 h. The optical density of each well was measured at 405 nm using a microplate reader. Each plate contained multiple wells of a given experimental condition and multiple control wells. The activity of caspase-3 was expressed in arbitrary absorbance units (absorbance at a wavelength of 405 nm).

### Statistical analysis

The Chi-square test was employed to analyze the positive rate of immunohistochemistric staining, and the correlation between pGSK-3β (*s*9) expression and MMR gene levels. Differences between different groups of HeLa cells in protein analysis, cell viability assays, and apoptosis studies were analyzed using ANOVA and a two-tailed *t*-test. The significance level was set at *P *< 0.05.

## Results

### Expression of PMS2 and pGSK-3β in human cervical carcinoma tissues

**Figure 1:**
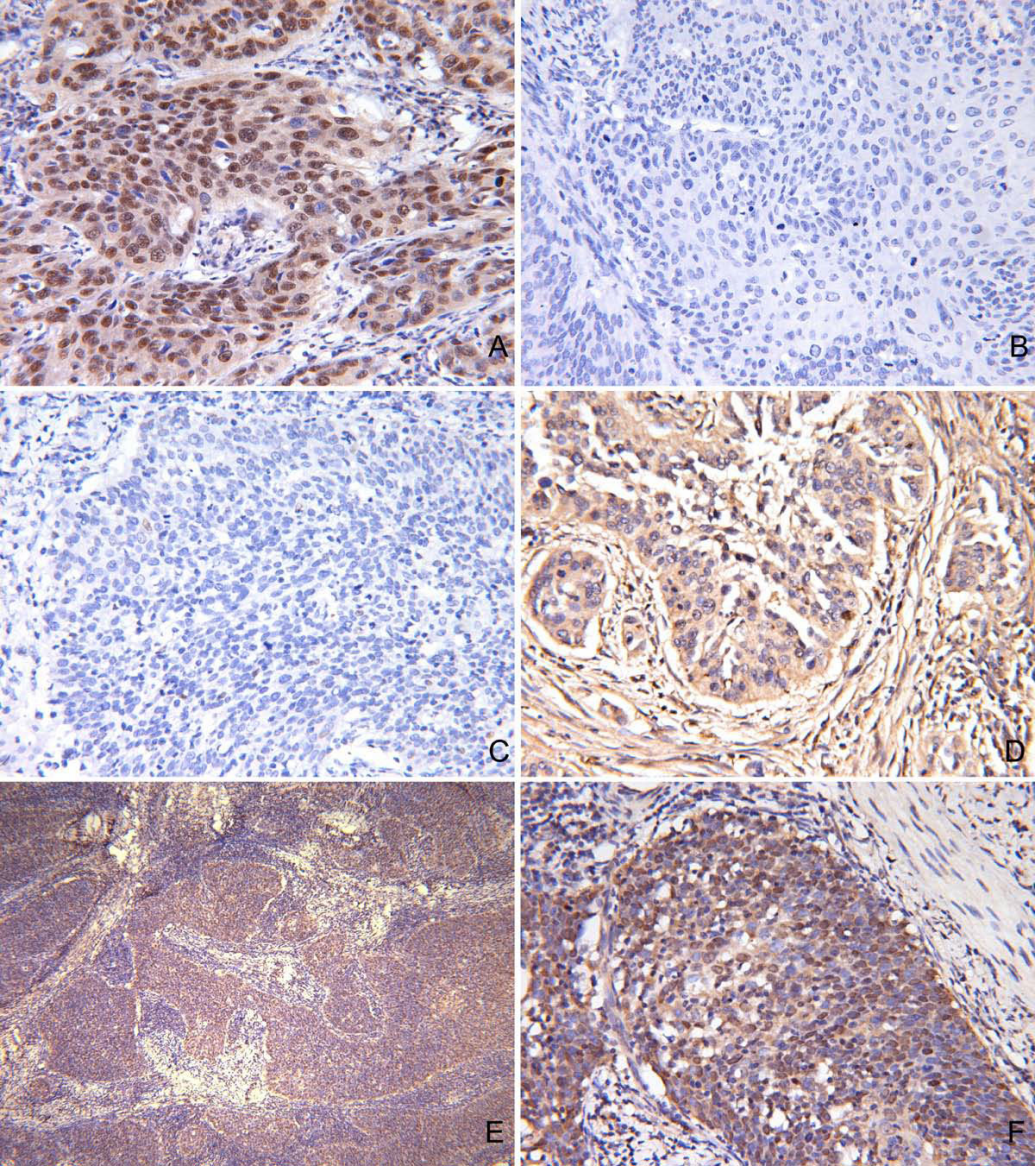
**Immunohistochemical staining of pGSK-3β, PMS2, MLH1, and MSH2 in human cervical carcinoma tissues**. Representative results of immunostainings were presented. (A) PMS2 positive stain in human cervical carcinoma tissues (× 400); (B) pGSK-3β negative stain in human cervical carcinoma tissues (× 400); (C) PMS2 negative stain in human cervical carcinoma tissues (× 400); (D) pGSK-3β positive stain in human cervical carcinoma tissues (× 400); (E) MLH1 positive stain in human cervical carcinoma tissues (× 200); (F), MSH2 positive stain in human cervical carcinoma tissues (× 400).

Few studies have described the clinical features of cases with PMS2 production in cervical carcinoma tissues up to now, but aberrant expression of MSH2 was shown to increase the risk in early stages of cervical carcinoma. To investigate the importance of the MMR genes (MSH2, PMS2) in cervical carcinoma and possible regulation by GSK-3β, we examined expression of MLH1, MSH2, PMS2, and pGSK-3β(*s*9) by immunohistochemistric staining. The clinical pathologic characteristics of specimens, including tumor size, lymph node status, tumor grade, distant metastasis and biomarker expression were obtained and are listed in Table 1. Of 78 tumor sections, 54 (69.2%) showed positive immunostaining for MLH1, 69 (88.5%) showed positive immunostaining for MSH2, 26 (33.3%) were positive for PMS2, and 48 (61.5%) were positive for pGSK-3β (*s*9). Representative examples of immunostaining slides are shown in Figure 1.

**Table 1:** Description of the patient population and biomarker expression

Characteristic	Patients (n = 78)
Age (Median ± SD)	47.2 ± 26.7
Histology	
Undifferentiated	51 (65.3%)
Differentiated	27 (34.7%)
Primary tumor stages	
T(1)	29 (37.2%)
T(2)	11 (14.1%)
T(3)	25 (32.1%)
T(4)	13 (16.6%)
Nodular metastasis	
Yes	22 (28.2%)
No	56 (71.8%)
Distant metastasis	
Yes	7 (8.9%)
No	71 (91.1%)

Because phosphorylation of *s*9 is known to inactivate GSK-3β kinase activity and can be used as a measurement of inactivation, we compared expression of MLH1, MSH2, and PMS2 with pGSK-3β(*s*9) level in cervical carcinoma. We found a statistically significant negative correlation between pGSK-3β(inactivation of GSK-3β) and PMS2 in cervical carcinoma (Table 2) (*P *= 0.001), while pGSK-3β did not show a statistically significant correlation with MLH1 and MSH2 immunoreactivity (data not shown). Our results suggested that the downregulation of PMS2 in cervical carcinoma tissues might be associated with pGSK-3β production and GSK-3β inactivation. Our results therefore have provided the possibility that GSK-3β is able to stabilize PMS2 production in human tissues.

**Table 2:** Correlation between pGSK-3β (*s*9) expression and PMS2 expression in cervical carcinoma patients

	pGSK-3β (+)	pGSK-3β (-)		
	No (%)	No (%)	*x*^2 ^value	*P *value
PMS2				
(-)	38	14	8.775	*P *= 0.001
(+)	10	16		

### Cisplatin inhibits cell viability of HeLa cells and increases PMS2 production

**Figure 2:**
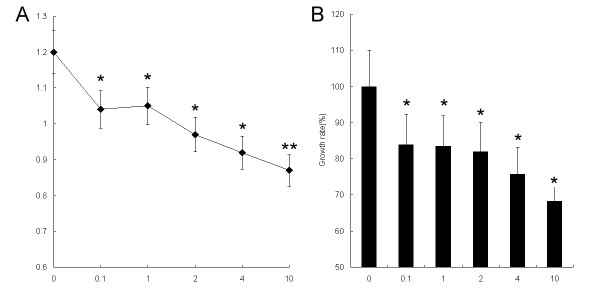
**Cell viability after cisplatin administration for 24 h in HeLa cells**. Treatment with different concentrations of cisplatin (from 0 to 10 μg/ml) for 24 h significantly decreased the cell growth of HeLa cells compared to the control, showing a concentration-dependent suppression. (A) Cell growth curve after different concentrations of cisplatin administration for 24 h; (B) Suppression of cell growth after different concentrations of cisplatin administration for 24 h. * *P *< 0.05, ** *P *< 0.01, compared to the control cells.

**Figure 3:**
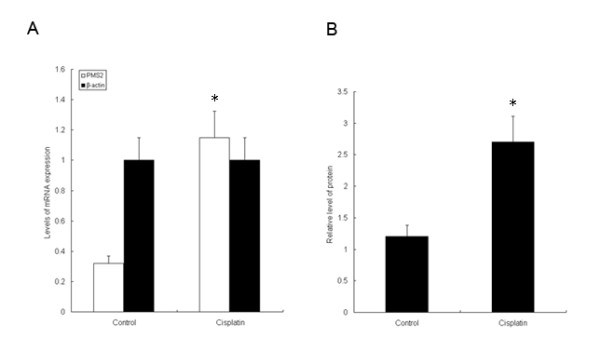
**Levels of PMS2 mRNA and protein in HeLa cells after cisplatin administration**. (A), PMS2 mRNA expression was significantly upregulated after treatment with cisplatin (10 μg/ml) for 24 h, as determined by semi-quantitative RT-PCR; (B), PMS2 protein level was significantly upregulated after treatment with cisplatin (10 μg/ml) for 24 h, as determined by immunoblotting. * *P *< 0.05, compared to the control cells.

It is well known that chemosensitization to chemotherapeutic agents usually is thought to result from apoptosis induction and functional MMR and drug resistance is associated with MMR deficiency, we thus postulated that treatment with cisplatin on HeLa cells may result in an MMR response and PMS2 upregulation. To determine the optimal concentration of cisplatin for evaluating PMS2 expression in the following step, we first investigated the effect of cisplatin on the growth of HeLa cells by MTT assay after treatment with cisplatin for 24 h. As illustrated in Figure 2, we found that treatment with different concentrations of cisplatin (from 0 to 10 μg/ml) for 24 h significantly decreased the cell growth of HeLa cells when compared to the control (*P *< 0.05), showing a concentration-dependent suppression. We therefore determined the optimal concentration of cisplatin as 10 μg/ml for further study on the basis of the growth curve of HeLa cells. We then examined the level of PMS2 protein in HeLa cells using western blotting after treatment with cisplatin (10 μg/ml) for 24 h. As illustrated in Figure 3, we observed the expression of PMS2 in HeLa cells is much low. The cisplatin treatment was shown to significantly increase the PMS2 production in HeLa cells, which was consistent with the mRNA expression. These results suggested that PSM2 was upregulated significantly at both the mRNA and protein level, and that it may contribute to the cisplatin-induced apoptosis in HeLa cells.

### Evidence indicates the interaction of GSK-3β and PMS2 in HeLa cells

**Figure 4:**
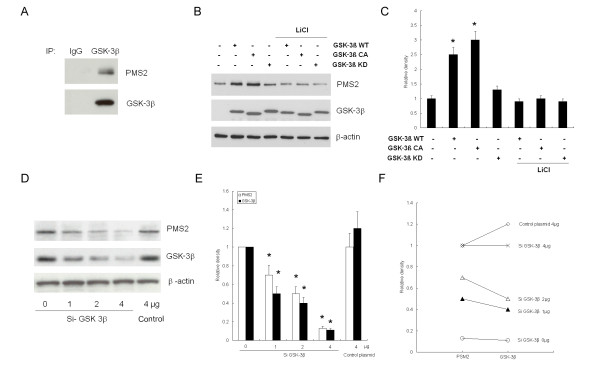
**Evidence indicates the interaction of GSK-3β and PMS2 in HeLa cells**. (A) Representative result of IP and immunobloting was presented after GSK-3β WT plasmid transfection. Interaction of GSK-3β and PMS2 in HeLa cells was suggested by using IP with an antibody to GSK-3β and immunoblotting with an antibody to PSM2; (B) Representative result of immunobloting was presented after GSK-3β plasmid transfection and Lithium administration; (C) GSK-3β significantly enhanced PMS2 production in HeLa cells, which was reversed by 20 nm of Lithium administration, * *P *< 0.05, compared to the control cells; ** *P *< 0.05, compared to the transfected cells; (D) Representative result of immunobloting was presented after GSK-3β plasmid transfection and RNA interference; (E) GSK-3β siRNA significantly reversed GSK-3β-enhanced PMS2 production in HeLa cells in a concentration-dependent fashion, * *P *< 0.05, compared to the control cells; (F) PMS2 protein was downregulated significantly after GSK-3β siRNA administration, showing a dose-dependent correlation (*r *= 0.792, *P *< 0.05).

Given that preliminary sequence analysis of PMS2 indicated that there exists a consensus motif of phosphorylation in the PSM2 gene which can be recognized and activated by GSK-3β, we hypothesized that GSK-3β is able to stabilize PSM2 by binding to the specific phosphorylation motif. To investigate the effect of GSK-3β on PMS2 production in HeLa cells, we transfected GSK-3β WT plasmid into HeLa cells and harvested the cells after 24 h for Co-IP and immunoblotting analysis. We evaluated the association between GSK-3β and PMS2 by using IP with an antibody to GSK-3β and immunoblotting with an antibody to PSM2. As illustrated in Figure 4A, we detected PSM2 production in the IP complex, indicating an interaction between GSK-3β and PMS2 in HeLa cells.

To further determine the effect of GSK-3β activity on PSM2 production in HeLa cells, we divided HeLa cells into three groups and transfected them with GSK-3β WT, GSK-3β CA, or GSK-3β KD plasmids and harvested the cells 24 h after for immunoblotting analysis. We then compared PMS2 production in the different transfection groups by immunoblotting analysis. As illustrated in Figure 4B, our results show that PMS2 production in the GSK-3β WT and GSK-3β CA groups was significantly higher than that in the GSK-3β KD group and control, indicating that GSK-3β WT and GSK-3β CA plasmids were able to stabilize PMS2 production in HeLa cells (*P *< 0.05).

**Figure 5:**
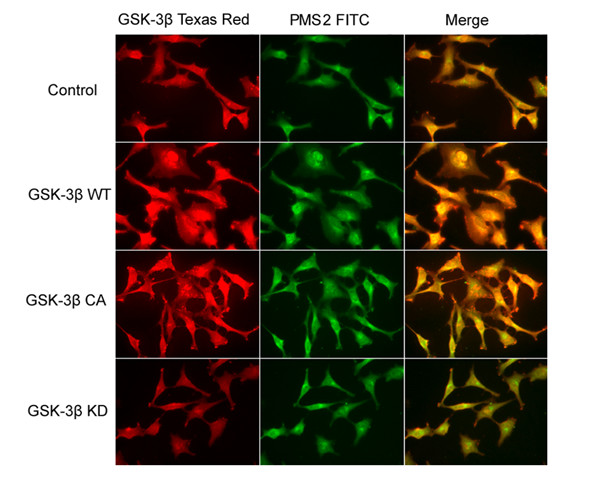
**Co-location of GSK-3β and PMS2 production in HeLa cells after GSK-3β transfection, as indicated by indirect immunofluorescence staining**. Representative results for GSK-3β and PMS2 immunostaining were presented (200×). Immunostaining of GSK-3β was cytoplasmic and partly nuclear in HeLa cells, while PMS2 was mainly located in the nucleus. Transfection of GSK-3β WT and GSK-3β CA but not GSK-3β KD increased the intensity of PMS2 expression in HeLa cells, compared to the control. GSK-3β was labeled with Texas Red-conjugated secondary antibody, while PMS2 was labeled with FITC-conjugated secondary antibody. The right lane indicated co-location of GSK-3β and PMS2 in the merged image in HeLa cells.

We then used LiCl, a specific inhibitor of GSK-3β, and GSK-3β siRNA to further evaluate the effect of blocking GSK-3β activity on PMS2 production in transfected HeLa cells. As illustrated in Figure 5C, we found that blocking GSK-3β activity with Lithium (20 mM) resulted in significantly downregulated PMS2 production in the GSK-3β WT and GSK-3β CA groups (*P *< 0.05), while there was no significant alteration of PMS2 production in the GSK-3β KD group after LiCl administration. We used GSK-3β WT-transfected HeLa cells as a control and found that PMS2 production was shown to be upregulated significantly in HeLa cells compared to non-transfected cells. Consistent results were observed in HeLa cells transfected with different concentrations of GSK-3β siRNA (1-4 μg) (Figure 4D, E). After GSK-3β siRNA administration, we observed that PMS2 protein levels were downregulated significantly compared to the control (*P *< 0.05), showing a dose-dependent correlation. We also examined the co-location of GSK-3β and PMS2 protein in HeLa cells by indirect immunofluorescence staining. As shown in Figure. 5, we found the immunostaining of GSK-3β was cytoplasmic and partly nuclear in HeLa cells, and PMS2 presented a similar distribution. Furthermore, transfection with GSK-3β WT and GSK-3β CA plasmids increased the intensity of both GSK-3β and PMS2 expression, compared to the control. Conversely, GSK-3β KD did not increase the intensity of either GSK-3β or PMS2 expression in HeLa cells. Together, these results provide evidence that GSK-3β is able to stabilize PMS2 production in HeLa cells.

### PMS2 transfection enhances chemosensitization of HeLa cells to cisplatin

**Figure 6:**
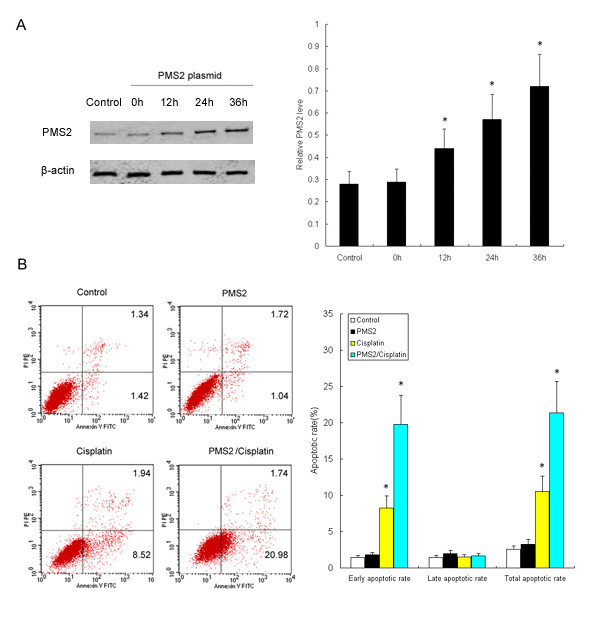
**PMS2 enhanced cisplatin-induced apoptosis in HeLa cells indicated by flow cytometric analysis**. HeLa cells were transfected with PMS2 plasmid (2 μg/mL) and followed by treatment with cisplatin for 0-36 h. (A) PMS2 production was demonstrated to be elevated in HeLa cells after transfection for 0-36 h at a dose of 2 μg/ml. Representative results of western blot were shown. (B) PMS2 transfection (24 h) significantly enhanced cisplatin-induced apoptosis in HeLa cells. Annexin^+^/PI^+ ^cells indicate late apoptosis cells and Annexin^+^/PI^- ^cells indicate early apoptosis cells. Representative results of apoptosis assay using the AnnexinV-FITC Apoptosis Detection Kit as described in Method Section was shown. The results are representative of two independent experiments. * *P *< 0.05 compared to the control cells.

To further characterize the importance of PMS2 in improving chemosensitization of cervical carcinoma, we analyzed the effects of PMS2 transfection on apoptosis of HeLa cells induced by cisplatin using flow cytometry. As shown in Figure. 6A, PMS2 production was demonstrated to be elevated in HeLa cells after transfection for 0-36 h at a dose of 2 μg/ml.

**Figure 7:**
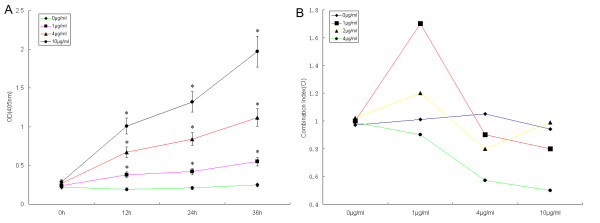
**PMS2 enhances caspase-3 activity in HeLa cells and improves cisplatin chemosensitivity**. Cells were transfected with the PMS2 plasmid and followed by a treatment with cisplatin for 0-36 h. (A), Caspase-3 activity was shown to be significantly increased after administration of the PMS2 plasmid (2 μg/ml) in cisplatin-treated HeLa cells. (B), Effect of combined PMS2 and cisplatin on HeLa cells. The combination index (CI) demonstrated a strong synergistic anti-tumor activity at lower doses of the PMS2 plasmid (2-4 μg/ml) and cisplatin (4-10 μg/ml) treatment in HeLa cells for 24 h.

For apoptotic analysis, HeLa cells were treated with PMS2 plasmid at a dose of 2 μg/ml or cisplatin at a concentration of 10 μg/ml, or a combination of the two. Populations of apoptotic cells were detected by fluorescence-activated cell sorting (FACS) analysis of Annexin V and PI stained cells at 24 h after the drug treatment. When compared with cells treated with PBS, PMS2, or cisplatin alone, we observed that the combination of PMS2 and cisplatin showed a significant increase of apoptosis in HeLa cells. As illustrated in Figure 6B, we found that the number of early apoptotic cells (Annexin V^+^/PI^- ^cells) and the number of total apoptotic cells (Annexin V^+ ^cells) were increased 2.4-fold and 2.1-fold (*P *< 0.05), respectively, when compared to the untreated cells. This indicated that transfection of PMS2 plasmid significantly enhanced cisplatin-induced apoptosis of HeLa cells, showing an enhanced chemotherapeutic effect. Consistently, caspase-3 activity was shown to be significantly increased after administration of the PMS2 plasmid in cisplatin-treated HeLa cells for 0-36 h (*P *< 0.05) (Figure 7A).

The effect of combined PMS2 transfection and cisplatin therapy on the HeLa cells was also evaluated in our cell system. The cells were first transfected with PMS2 plasmid (0-4 μg/ml) and then exposed to cisplatin at various concentrations (0-10 μg/ml). Cells treated with empty plasmid were used as the control. Cell viability was determined at 24 h after treatment by using an MTT assay. We demonstrated a strong synergistic anti-tumor activity at the lower doses of the PMS2 plasmid (2-4 μg/ml) and cisplatin (4-10 μg/ml) in HeLa cells by CI (Figure 7B). The results therefore confirmed that PMS2 transfection improved chemosensitization of cisplatin on HeLa cells.

## Discussion

The significance of MMR in the development of cervical carcinoma has not been well documented, and few data are available. It is known that the role of the DNA MMR system is to maintain DNA replication fidelity by repairing DNA mismatches and insertion/deletion loops acquired during DNA replication [19]. This process is highly conserved, from *Escherichia coli *to mammals. PMS2 is a major component of the DNA mismatch repair system. However, unlike other components, PMS2 is highly unstable and germline PMS2 mutations are rarely reported. PMS2 has recently emerged as an attractive target due to its low rate of mutation and unstabilization [20,21], raising the possibility that certain types of exogenous DNA-damaging agents may rapidly degrade PMS2 production and disrupt the MMR system for facilitating carcinogenesis, and the rapid induction and degradation of PMS2 may be essential for the balance between cell survival and death as a sensor of environmental changes. In the present study, we found a significant PMS2 downregulation in human cervical carcinoma tissue, similar to the results found in other cancers. Moreover, we provide the first evidence that increased expression of phosphorylated GSK-3β (*s*9) (GSK-3β inactivation) is negatively correlated with PMS2 expression in cervical carcinoma and that GSK-3β is able to stabilize PMS2 production in HeLa cells, implicating a novel target to explore in the carcinogenesis and chemotherapy enhancement of human cervical carcinoma.

Irrespective of gene mutations, varied levels of MMR proteins have recently been reported in a variety of tumors, such as colon cancer, lung cancer, and breast cancer, that were responsible for the prognosis [22]. A plausible explanation is that the disregulated DNA replication system is controlled and that no abnormal transcription of MMR protein occurs in normal cells, whereas in the abnormal tumor cells, MMR genes are rapidly activated and upregulated. Therefore, the loss of MMR activity due to rapid degradation may lead to less efficient gene repair, chemo-resistance and poor prognosis. To investigate the importance of MMR protein in this study, we firstly examined the expression of PMS2, MSH2 and MLH1 in cervical carcinoma by immunohistochemical staining. We found a significant downregulation of PMS2 in cervical carcinoma comparing to other protein. Furthermore, we detected PMS2 expression in cisplatin-induced HeLa cells and found a significant upregulation of mRNA and protein, compared to the control. The decrease of PMS2 activity in tissue and the induction of PMS production in HeLa cells suggested that the PMS2 protein may play a more important role in the development of cervical carcinoma.

Interestingly, we also found that PMS2 expression was negatively correlated with GSK-3β inactivation in tumor tissues, indicating that activated GSK-3β may stabilize PMS2 production directly. It is now clear that GSK-3β functions in diverse cellular processes including proliferation, differentiation, survival, neoplastic transformation and tumor development [23]. The downstream targets of GSK-3β include some well-recognized genes such as Axin1, c-myc, β-catenin, snail, and MCL-1, which have a close association with carcinogenesis. Interestingly, all of them were found to share a similarity in structure by possessing the common motif of S/T XXX S/T. In our preliminary study, we found a consensus motif of VSSSH GPSDP TDRAE in PMS2 by gene sequencing. This finding indicated that PMS2 may be recognized by GSK-3β, and additional studies will be required to understand the interaction of GSK-3β and PMS2 in cervical carcinoma. This finding, together with the immunohistochemical results, raised the possibility that GSK-3β may be able to stabilize PSM2 production in cervical carcinoma. To prove our hypothesis, we transfected the GSK-3β gene into HeLa cells and evaluated the interaction of GSK-3β and PMS2 by using IP with an antibody to GSK-3β and immunoblotting with an antibody to PSM2. Surprisingly, we detected the PSM2 production in the IP complex, which provided the convincing evidence that GSK-3β is able to bind to PMS2 protein.

GSK-3β has become an important area of investigation as a key component of the Wnt signaling pathway. Unlike most protein kinases, GSK-3β is constitutively active in resting cells, and it undergoes a rapid and transient inhibition in response to a number of external signals. To investigate the interaction of GSK-3β and PMS2 in HeLa cells, we evaluated the effects of blocking GSK-3β activity using a GSK-3β KD plasmid, GSK-3β siRNA, and a specific inhibitor, LiCl, on the PMS2 production in HeLa cells. Consistently, our results showed that PMS2 production was downregulated in HeLa cells. And we observed a concentration-dependent reduction of PMS2 production in HeLa cells after administration of GSK-3β siRNA and Lithium. Furthermore, we observed co-location of GSK-3β and PMS2 protein in HeLa cells by indirect immunofluorescence staining, which was enhanced by transfection with GSK-3β WT and GSK-3β CA, but not GSK-3β KD. Taken together, these results provided evidence that GSK-3β is able to stabilize PMS2 production in HeLa cells.

Furthermore, it is very important to note that cervical cancer affects women at a higher incidence and a younger age, with the increased prevalence of human papillomavirus and alteration of socioeconimic condition [24], and prolonged cisplatin treatment appears to induce multiple mechanisms of tumor resistance [25]. On the other hand, the MMR system is also implicated in the cellular response to DNA damage, and MMR deficiency was thought to be responsible for drug resistance during chemotherapy in a variety of cancers [26-28]. PMS2 are able to activate cell cycle checkpoints and apoptosis in response to persistent DNA damage [29]. It is shown that PMS2 is required for cisplatin-induced activation of p73, a member of the p53 family of transcription factors with proapoptotic activity [30]. However, the significance of PMS2 deficiency in drug resistance is controversial. Gibson et al. showed that dysregulation of PMS2 gene expression can disrupt MMR function in mammalian cells and establish an additional carcinogenic mechanism by which cells can develop genetic instability and acquire resistance to cytotoxic cancer therapies [31]. In addition, Fedier et al. investigated the effects of loss of PMS2 on the sensitivity to a panel of widely used anticancer agents in E1A/Ha-Ras-transformed p53-null mouse fibroblasts either proficient or deficient in PMS2 and found that lack of the PMS2 gene is associated with an increased sensitivity to some types of anticancer agents such as cisplatin, oxaliplatin, and gemcitabine [32]. To investigate whether loss of function of PMS2 plays a role in drug resistance in HeLa cells or not, we examined the effects of PMS2 transfection on apoptosis of HeLa cells induced by cisplatin using flow cytometry. We found that apoptotic cells and caspase-3 activity increased significantly, compared to the untreated cells. We also demonstrated a strong synergistic anti-tumor activity at lower doses of the PMS2 plasmid and cisplatin in HeLa cells by CI analysis. We therefore concluded that PMS2 upregulation significantly enhanced cisplatin-associated chemosensitivity, which may be useful for the treatment of cervical carcinoma. On the other hand, we confessed that our study contained some insufficiencies to investigate the associated mechanisms underlying MMR-deficiency in cervical carcinoma. The detailed mechanism underlying MMR-deficiency in cervical carcinoma is still not fully understood and other MMR components (MSH2, MLH1, MSH6) may be functional related with PMS2, which requires further study.

## Conclusion

Our results provide the preliminary evidence that GSK-3β stabilized PMS2 production, which may play an important role in the carcinogenesis and chemotherapy of cervical carcinoma. This newly identified mechanism will be helpful in better understanding tumor development and in designing a novel therapeutic strategy for the treatment of human cervical carcinoma.

## Abbreviations

GSK-3βL: glycogen synthase kinase 3β; siRNA: small interference RNA; RT-PCR: transcription polymerase chain reaction; MTT: 3-(4,5-Dimethylthiazol-2-yl)-2,5-diphenyltetrazolium bromide; cisplatin: cis-diammine-dichloroplatinum; DMSO: dimethyl sulfoxide; LiCl: lithium chloride.

## Competing interests

The authors declare that they have no competing interests.

## Authors' contributions

YZ and YS performed the entire experiment. SW and BD participated in partial experiment (gene transfection and flow cytometric analysis). ZW performed the statistic analysis. HL designed the study and prepared the manuscript. All authors read and approved the final manuscript.

## Pre-publication history

The pre-publication history for this paper can be accessed here:

http://www.biomedcentral.com/1471-2407/10/58/prepub
